# Rare earth element nucleosynthetic anomalies and dust transport in the protoplanetary disk

**DOI:** 10.1126/sciadv.adv3148

**Published:** 2025-07-09

**Authors:** Justin Y. Hu, François L. H. Tissot, Ren T. C. Marquez, Oliver Shorttle, Cathie J. Clarke, Andrew D. Sellek, Nicolas Dauphas, Bruce L. A. Charlier, Ingo Leya, Reika Yokochi, Thomas J. Ireland, Helen M. Williams

**Affiliations:** ^1^ Origins Laboratory, Department of the Geophysical Sciences and Enrico Fermi Institute, The University of Chicago, Chicago, IL 60637, USA.; ^2^ Department of Earth Sciences, University of Cambridge, Cambridge CB2 3EQ, UK.; ^3^ The Isotoparium, Division of Geological and Planetary Sciences, California Institute of Technology, Pasadena, CA 91125, USA.; ^4^ Buseck Center for Meteorite Studies, Arizona State University, Tempe, AZ 85281, USA.; ^5^ Institute of Astronomy, University of Cambridge, Cambridge CB3 0HA, UK.; ^6^ Leiden Observatory, Leiden University, Leiden 2300 RA, Netherlands.; ^7^ School of Geography, Earth and Environmental Sciences, Victoria University of Wellington, Wellington 6140, New Zealand.; ^8^ Space Sciences and Planetology, University of Bern, Bern 3012, Switzerland.; ^9^ Cambridge Isotope Laboratories, Inc., Tewksbury, MA 01876, USA.

## Abstract

The size, density, and chemical characteristics of solar system bodies have been shaped by material transport during the protoplanetary disk stage. This includes transport from the inner to outer solar system of refractory dust grains that carry nucleosynthetic anomalies. Here, we show that rare earth element (REE) isotopes in fine-grained calcium-aluminum–rich inclusions (CAIs) display anomalies stemming from incomplete mixing of *r*-, *s*-, and *p*-process nucleosynthesis. The data points define two correlations, which are best explained by mixing between three isotopic reservoirs in two successive stages, one of which involved a variable admixture of a *p*-process component. We propose that CAI precursors formed in the inner solar system and were subsequently transported by FU Orionis outbursts from the disk to the envelope where they mixed with an isotopically distinct reservoir before settling on the midplane.

## Teaser

Isotopic anomalies of REEs in fg-CAIs reveal incomplete solar system mixing, potentially linked to FU Orionis outbursts.

## INTRODUCTION

The distribution of materials during the protoplanetary disk stage was primarily shaped by processes affecting radial transport within the disk, along with those that remove material from the disk, potentially returning it to the disk at larger radii. Gas in the disk is subject to each of these effects, including inward accretion flows in the disk driven by a range of (magneto-)hydrodynamical effects and outflows in the form of jets and wide-angle winds, which may be either photoevaporative or magnetic in nature. The complex interplay between these processes can lead to time-dependent episodes of ejection/accretion. Dust, conversely, responds to gas dynamics via drag forces. For larger grains, the dynamical coupling between dust and gas is imperfect and partial coupling leads to dust radial drift in disks, as well as selective entrainment of small grains in winds/outflows. For reviews of these topics, see (*1*–*4*). Because different species reside in solid and vapor forms at different radii within the disk, this transport profoundly influenced the size, density, and chemical and isotopic characteristics of solar system (SS) objects.

Isotopic anomalies have been observed at all scales in the SS, from nanodiamonds a few thousand of atoms in size to regions of the protoplanetary disk spanning several astronomical units (AUs) (*5*). Although these isotopic anomalies are often smaller in magnitude compared to the more pervasive mass-dependent isotope fractionation effects observed in meteorites, they can survive chemical processes and are valuable for tracing the origins and transport of material within the protoplanetary disk.

Nucleosynthetic isotopic anomalies, which result from incomplete mixing of materials produced at different nucleosynthesis sites, offer a way to track material transport in the early SS (*6*). Heterogeneity in nucleosynthetic anomalies has been seen throughout the SS among (i) noncarbonaceous chondrite (NC) clan materials thought to sample the inner SS, (ii) carbonaceous chondrite (CC) clan materials thought to sample the SS beyond the snowline, and (iii) CI reservoir represented by CI chondrites and asteroid Ryugu (*7*). This large-scale distribution of nucleosynthetic anomalies must have been shaped by mixing processes in the protoplanetary disk, indirectly recording aspects of disk dynamics that remain debated. As discussed by Dauphas *et al.* (*8*), the two possible scenarios for the origin of planetary-scale isotopic heterogeneities are mapping of nucleosynthetic anomalies from the protosolar molecular cloud core directly onto the protoplanetary disk (*5*, *9*) or creation of isotopic anomalies from an originally isotopically homogeneous phase by grain size sorting or gas-dust decoupling.

Calcium-aluminum–rich inclusions (CAIs), composed of highly refractory minerals and recognized as the oldest dated materials formed in the SS (*10*, *11*), are known to carry nucleosynthetic anomalies originating from the *p*-, *s*-, and *r*-processes (*12*–*14*), as well as those affecting iron-peak elements such as Ca and Ti (*5*). The extremely high formation temperatures (*15*), solar O isotopic composition (*16*), and the presence of cosmic-ray spallation–induced ^10^Be isotopic anomalies (*17*) indicate that CAIs were likely formed within 1 AU of the protosun (*18*). However, the widespread presence of CAIs in CCs and some comets suggests that these refractory mineral assemblages were then transported from the inner SS where they formed to the outer regions during the solar nebula stage. The presence of various nucleosynthetic anomalies and the history of transport through the protoplanetary disk make CAIs ideal targets to study early SS mixing.

Fine-grained CAIs (fg-CAIs) are the most common type of refractory inclusion found in chondrites (*10*, *19*). Their texture indicates that they were never molten since their initial condensation. A substantial portion of fg-CAIs preserves highly fractionated rare earth element (REE) patterns, known as group II patterns, characterized by the uniform enrichment of moderately refractory REEs (La-Sm and Tm) and depletions in both the most volatile (Eu and Yb) and refractory REEs (Gd-Er and Lu), relative to solar composition (*20*, *21*). The preferential enrichment in light REE (LREE) isotopes found in the most refractory REEs in the fg-CAIs with group II REE patterns indicates that the fg-CAIs have experienced substantial kinetic evaporation (*22*). The evaporation could have been caused by intense heating related to outbursts triggered by FU Orionis and Ex Lupi events near the protosun (*22*), which can have a profound influence on material transport in the protoplanetary disk.

Here, we report isotopic anomalies of Nd, Sm, Gd, Dy, Er, and Yb in a group of well-characterized fg-CAIs, which have experienced different thermal histories compared to the coarse-grained CAIs (cg-CAIs) (*10*). While isotopic anomalies of REEs have been measured previously in CAIs (*23*, *24*), these studies focused on cg-CAIs [figure 3 in (*25*)]. Only a few fg-CAIs were analyzed in (*23*, *24*), and these show less fractionated group II patterns compared to the fg-CAIs analyzed here. In our fg-CAI study, we correct for cosmic-ray exposure (CRE) effects known to influence isotopes with large neutron capture cross sections such as ^149^Sm, ^155^Gd, and ^157^Gd. We evaluate the relationships between REE nucleosynthetic anomalies and compare them to Ti and Sr isotopic anomalies that were measured in the same CAIs, which shed light on the protoplanetary disk processes that shaped the chemical and isotopic compositions of planetary materials.

## RESULTS

**Fig. 1.**
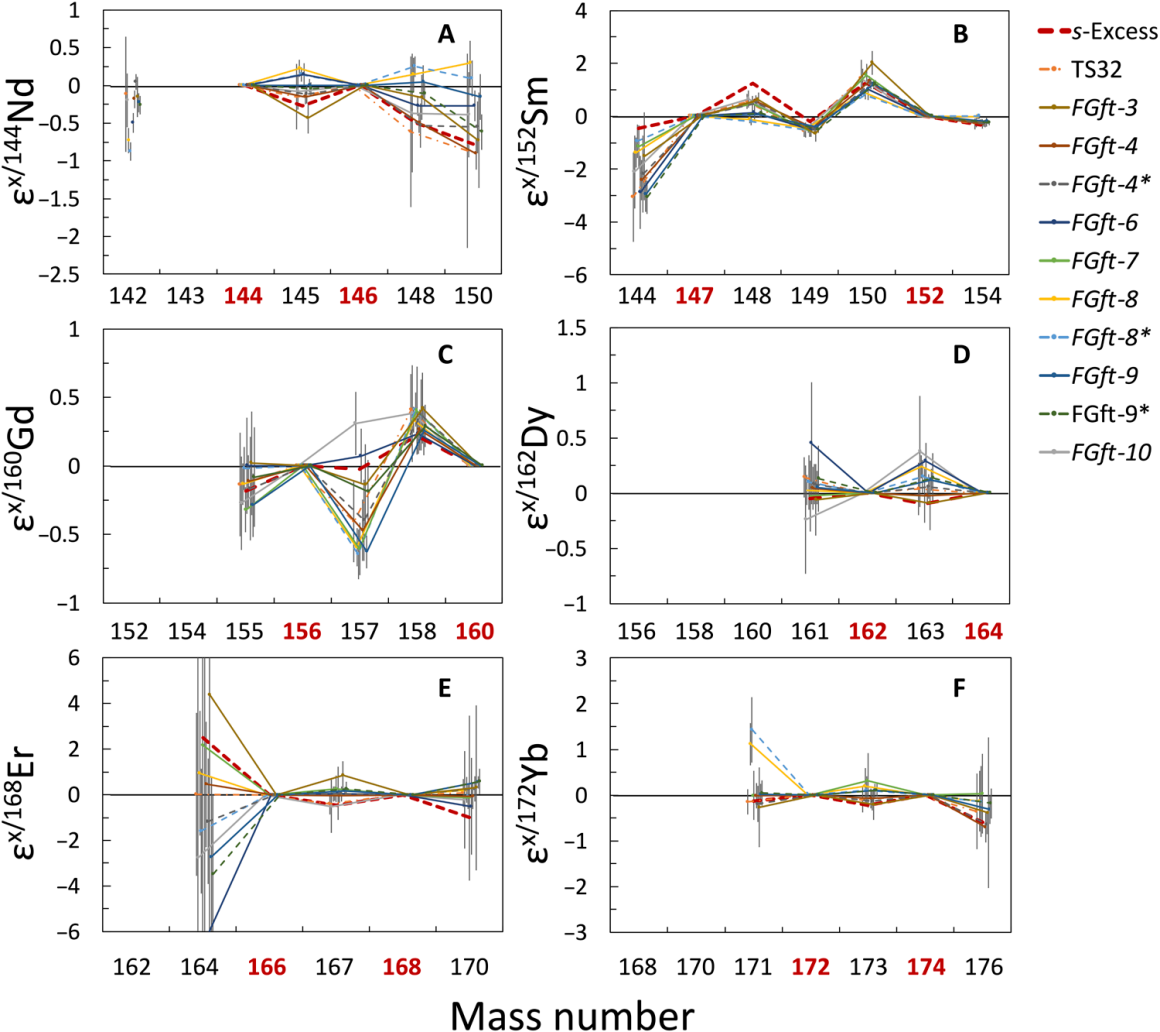
Internally normalized REE isotope patterns for the CAIs. (**A** to **F**) Isotope patterns for Nd (A), Sm (B), Gd (C), Dy (D), Er (E), and Yb (F) in the CAIs. All the isotopic anomalies are normalized to terrestrial geostandard BCR-2. The bold red numbers indicate the isotopes used for internal normalization. Samples marked with “*” are replicates. The red dashed lines are modeled *s*-excesses obtained by adding *s*-process abundances to cosmic (solar) abundances (*28*) using equation A10 of (*32*). The magnitude of the modeled *s*-excess is arbitrary; what matters here is the pattern.

Details of the methodology for REE purification and isotopic analyses are reported in (*22*). Note that all isotopes of the REEs are not reported because of insufficient precision as a result of the extremely low natural abundance of some isotopes. Isotopic anomalies are isotopic variations that cannot be accounted for by mass-dependent fractionation (MDF) and radioactive decay. Isotopic variations after internal normalization to correct for MDF are presented for Nd, Sm, Gd, Dy, Er, and Yb in tables S1 and S2 and plotted in Fig. 1. Isotopic variations are reported in ε-notation, the deviation in parts per 10,000 of the internally normalized isotopic ratios relative to a terrestrial standard. The measurements were bracketed by the OL-REE standards, but because these were industrially concentrated, which can affect isotopic ratios, we report the ε-values relative to terrestrial geostandard BCR-2 passed through the same column procedure, with the uncertainties propagated accordingly. Three CAIs (*FGft*-4, *FGft*-8, and *FGft*-9) were processed twice, starting from a single digestion, to assess the reproducibility of our measurements. Duplicates are indicated by thick tie lines in all figures. There is good agreement between the duplicate analyses, apart from ε^170/172^Yb in *FGft*-8.

Systematic negative isotopic variations are observed for ε^148/144^Nd and ε^150/144^Nd for the fg-CAIs after normalizing to ^146^Nd/^144^Nd = 0.7219 (Fig. 1A). The patterns for Sm isotopic variations in the fg-CAIs show resolved positive isotopic shifts for ^148^Sm and ^150^Sm, pronounced negative isotopic shifts for ^144^Sm, and subtle negative anomalies for ^149^Sm and ^154^Sm after normalization to ^147^Sm/^152^Sm = 0.56081 (Fig. 1B). Systematically negative ^176^Yb isotopic shifts were found after normalization to ^174^Yb/^172^Yb = 1.4772 (Fig. 1F). Similar Nd, Sm, and Yb isotopic variations have been previously reported for cg- and fg-CAIs in (*23*, *24*).

Gadolinium isotopes are normalized to (^155^Gd + ^156^Gd)/^160^Gd = 1.6129 to account for the depletion of ^155^Gd and enrichment of ^156^Gd induced by the neutron capture reaction ^155^Gd(n, γ)^156^Gd caused by CRE (*26*). The fg-CAIs broadly show negative isotopic shifts for ^157^Gd and positive isotopic shifts for ^158^Gd relative to the isotope standard OL-Gd and geostandard BCR-2 after normalization (Fig. 1C). No isotopic shifts of ^161^Dy and ^163^Dy were found after normalization to ^164^Dy/^162^Dy = 1.01237 (Fig. 1D). No systematic Er isotopic shifts were found after normalization to ^166^Er/^168^Er = 1.24140 (Fig. 1E). Shollenberger *et al.* (*24*) also analyzed Er isotopes in CAIs and found no resolvable Er isotopic anomaly [figure 3 in (*24*)].

The effect of cosmic-ray irradiation is discussed in detail in the “Correlations of anomalies of REE *r*/*s*-isotopes in CAIs” section, but overall, the isotopic anomaly patterns of Nd, Sm, and Yb observed in our study indicate that the fg-CAIs studied here have *r*-process deficits or *s*-process excesses similar to those documented in mainstream presolar SiC grains (*27*) that are believed to have condensed in the outflows of low-mass asymptotic giant branch (AGB) stars (*28*).

If chemical processes responsible for creating MDF were involved in fractionating the carriers of isotopic anomalies, one might expect correlations between isotopic anomalies and MDFs (*29*). Davis *et al.* (*29*) found that refractory inclusions with the highest ε^50/47^Ti values were the ones that showed the most dispersion in their Ti MDF. FUN CAIs and hibonite grains are the materials with the largest isotopic anomalies for Ti (outside of presolar grains), and they are also the ones that display the strongest Ti MDF reflecting thermal processing. For the fg-CAIs analyzed in this study, no relationships are observed between dispersion in MDF and the size of isotopic anomalies for Ti, Sr, Sm, and Er isotopes (fig. S1); thus, the isotopic anomalies observed are unlikely to be caused by the chemical process that induced MDFs.

## DISCUSSION

### Cosmic-ray irradiation on Sm and Gd isotopes

Galactic cosmic-ray protons interact with nuclei in chondrite parent bodies, generating high-energy neutrons that gradually slow down as they travel through the material, eventually reaching thermal levels (*26*, *30*, *31*). The isotopes ^149^Sm, ^155^Gd, and ^157^Gd have exceptionally large resonance integral (3433 barns for ^149^Sm from Evaluated Nuclear Data File ENDF/B-VIII) or thermal neutron capture cross sections (40,511.7 barns for ^149^Sm, 60,792 barns for ^155^Gd, and 253,299 barns for ^157^Gd from ENDF/B-VIII) and so are the most likely to be affected by CRE effects through neutron capture reactions ^149^Sm(n, γ)^150^Sm, ^155^Gd(n, γ)^156^Gd, and ^157^Gd(n, γ)^158^Gd, although the neutron capture reaction of ^155^Gd to ^156^Gd does not influence our results because the data are internally normalized to a fixed (^155^Gd + ^156^Gd)/^160^Gd ratio.

**Fig. 2.**
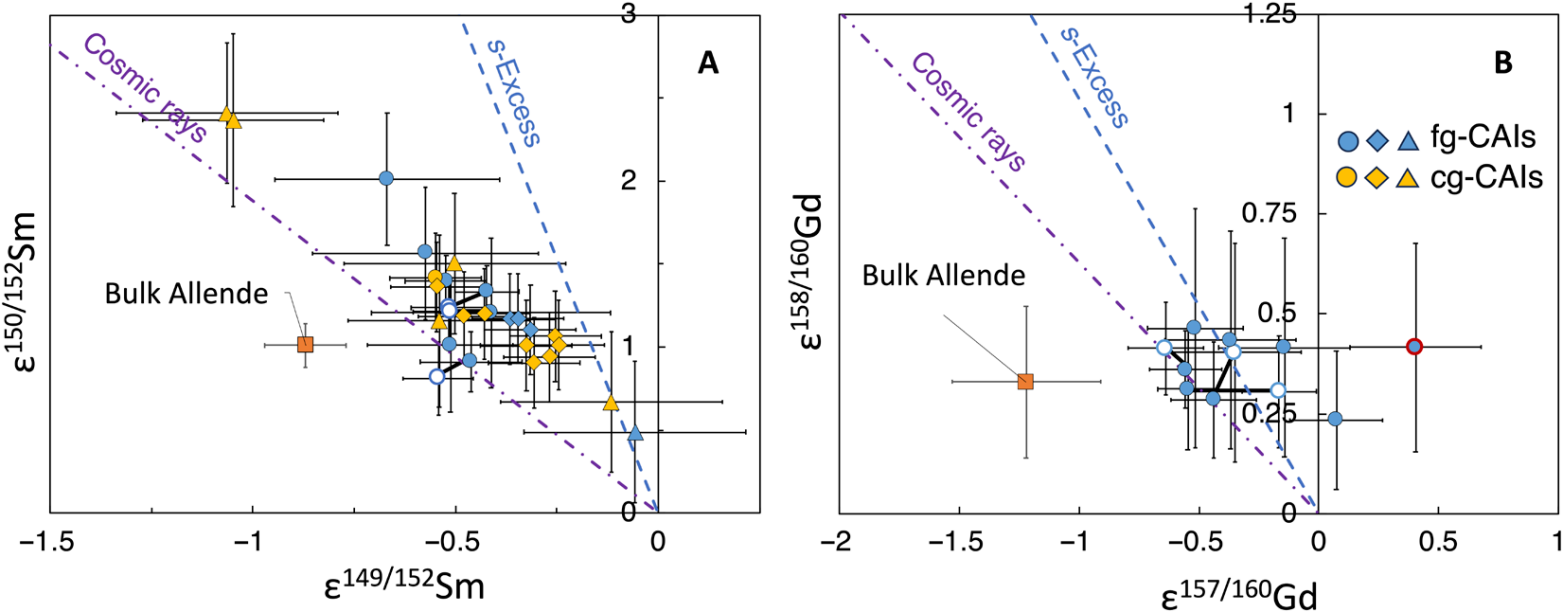
Isotope variations of Sm and Gd induced by CRE. (**A** and **B**) Plots of ε^150/152^Sm versus ε^149/152^Sm (A) and ε^158/160^Gd versus ε^157/160^Gd (B) for fg-CAIs (blue) and cg-CAIs (yellow). The open circles connected by thick lines are replicate analyses of CAIs *FGft*-4, *FGft*-8, and *FGft*-9. These open symbols correspond to three replicate analyses done using smaller sample quantities than the filled symbols, and the latter are therefore more reliable. Circles are from this study, and diamonds are from (*23*). Triangles are CAIs from non-Allende CV and CK chondrites in (*35*, *49*). The outlier in (B) (red outline) is fg-CAI *FGft-*10. All the isotopic anomalies are normalized to terrestrial geostandard BCR-2. Linear regressions forced through the origin point calculated exclusively from the fg-CAI yield slopes of −2.67 in (A) and −0.73 in (B). Purple dash-dot lines are lines expected for cosmogenic effects, and blue dashed lines are for nucleosynthetic anomalies caused by *s*-excesses (the isotopes depicted here have no *p*-process component).

**Fig. 3.**
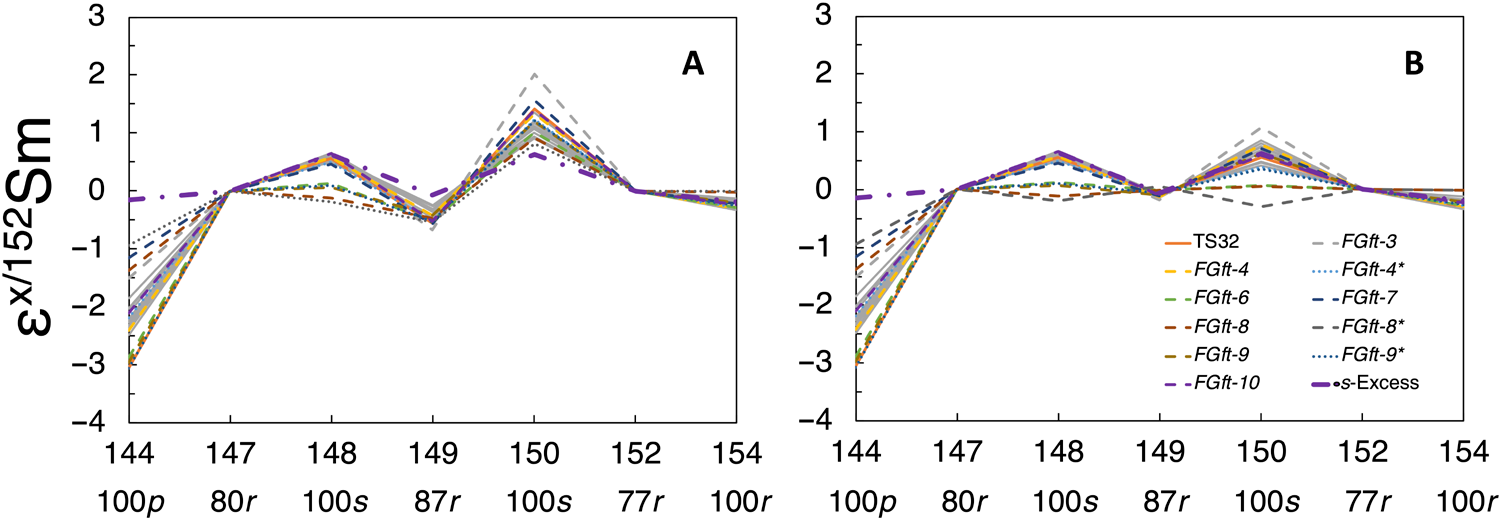
Samarium isotopic anomaly patterns in the CAIs. (**A** and **B**) Isotopic anomaly patterns before and after correction of the neutron capture effects caused by CRE by decomposing nucleosynthetic and cosmogenic contributions on ^149^Sm and ^150^Sm using the slopes in Fig. 2. The thick purple dash-dot line is a model fit of *s*-excess of arbitrary magnitude. The thin gray lines are from (*23*). All the patterns are normalized to the isotopic anomalies of terrestrial geostandard BCR-2. The dominant nucleosynthetic processes responsible for the production of each nuclide are indicated as percentages of the total below each isotope label. For all isotopes, when the contribution is not 100%, the remainder is either *s* or *r* (there are no mixed *s* + *p* or *r* + *p* isotopes).

We plot ε^150/152^Sm versus ε^149/152^Sm and ε^158/160^Gd versus ε^157/160^Gd in Fig. 2, as these isotope signatures are expected to define linear correlations with slopes close to −1.88 and −0.63, respectively, if the CAIs were affected by CRE effects alone. The CAIs show a negative correlation between ε^150/152^Sm and ε^149/152^Sm with a slope of −2.67 ± 0.38 after forcing the regression line to pass through the origin. The ε^158/160^Gd and ε^157/160^Gd values of the fg-CAIs tend to cluster and show a weak correlation with a slope of −0.73 ± 0.28 for a regression line passing through the origin. The difference between the slopes predicted for neutron capture effects and the measured slopes is likely due to isotopic anomalies of *r*/*s*-process origin, which are expected to give slopes of −6.13 and −1.04 for Sm and Gd isotopes, respectively (*28*, *32*, *33*). Nucleosynthetic anomalies will introduce scatter and make the slopes appear more negative than predicted for cosmic-ray effects, as is observed in both our Sm and Gd isotope data. The Sm data were corrected for the presence of cosmogenic effects by separating the nucleosynthetic and cosmogenic contributions on ^149^Sm and ^150^Sm on the basis of their expected slopes (Fig. 3). Specifically, the ratios of ^150^Sm/^149^Sm were decomposed by modeling the mixing of two components: one with a slope of −1.88 (cosmogenic) and the other with a slope of −6.13 (nucleosynthetic) for ^150^Sm/^149^Sm (*34*).

Correcting for CRE-induced Sm and Gd isotopic variations is crucial for identifying the source of isotopic anomalies in the *p*-isotope ^144^Sm, which is among the very few *p*-isotopes that are abundant enough to be measured with good precision. Brennecka *et al.* (*23*) normalized their Sm isotope data to a fixed ^148^Sm/^150^Sm ratio of 1.52370, but ^150^Sm can be produced by interaction between ^149^Sm and CRE-induced secondary neutrons. Normalization using ^150^Sm in the presence of CRE effects will therefore complicate the interpretation of Sm isotopic variations, particularly for Sm isotopes of the lowest (^144^Sm and ^147^Sm) and highest (^152^Sm and ^154^Sm) masses. Brennecka *et al.* (*23*) considered this issue but assumed that it was not significant (*35*). However, if Sm isotopic anomalies are instead normalized to ^147^Sm/^152^Sm = 0.56081 such that neither ^149^Sm nor ^150^Sm is used in the calculation of internally normalized ratio, we can more readily evaluate the origin of ε^144/152^Sm anomalies by comparing the data with model predictions involving variations in the contributions of different nucleosynthetic processes (*8*).

### Confirming the presence of *p*-isotope anomaly in ε^144/152^Sm

**Fig. 4.**
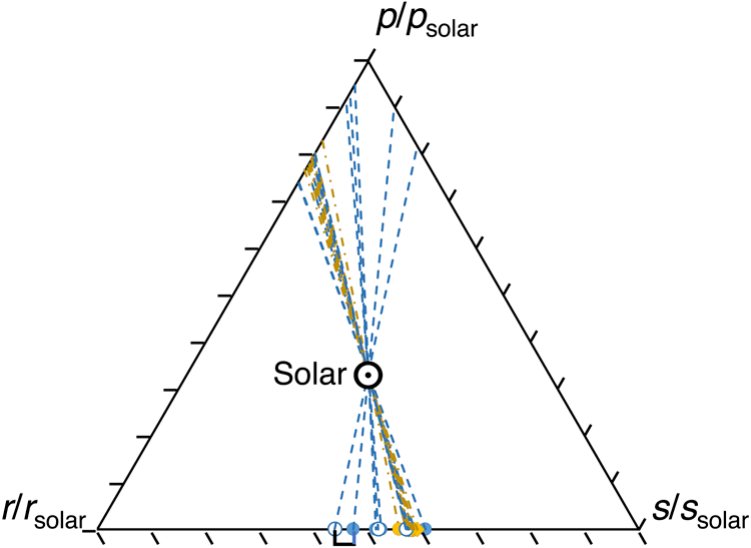
Ternary diagram of nucleosynthetic end-members (*28*) that need to be admixed with solar composition to explain Sm isotope patterns in the CAIs. See details in (*5*) and the Supplementary Materials. Blue circles and dashed lines are for fg-CAIs, and yellow circles and dash-dot lines are for cg-CAIs. Circles are from CAIs analyzed in this study, with the open symbols being replicates (*FGft*-4, *FGft*-8, and *FGft*-9; the replicates are connected with thick lines, but only one is visible because the model end-member compositions overlap for the other two replicates). Diamonds are from (*23*).

In addition to ^144^Sm, the fg-CAIs also show isotopic anomalies in another *p*-process isotope ^84^Sr (*36*). Isotopic variations in ε^144/152^Sm and ε^84/86^Sr could potentially be caused by variations in *s*- and *r*-isotopes as these are involved in the calculation of ε-values for these ratios through internal normalization. The isotopic anomalies of Sm can be decomposed in a ternary diagram of *p*-, *s*-, and *r*-contributions [figure 11 in (*5*); also see the Supplementary Materials for the mathematical approach used in this decomposition], which shows that the isotopic variation seen in CAIs is primarily controlled by a significant deficit of *p*-process material and a small excess of *s*-process material (Fig. 4).

### Correlations of anomalies of REE *r*/*s*-isotopes in CAIs

**Fig. 5.**
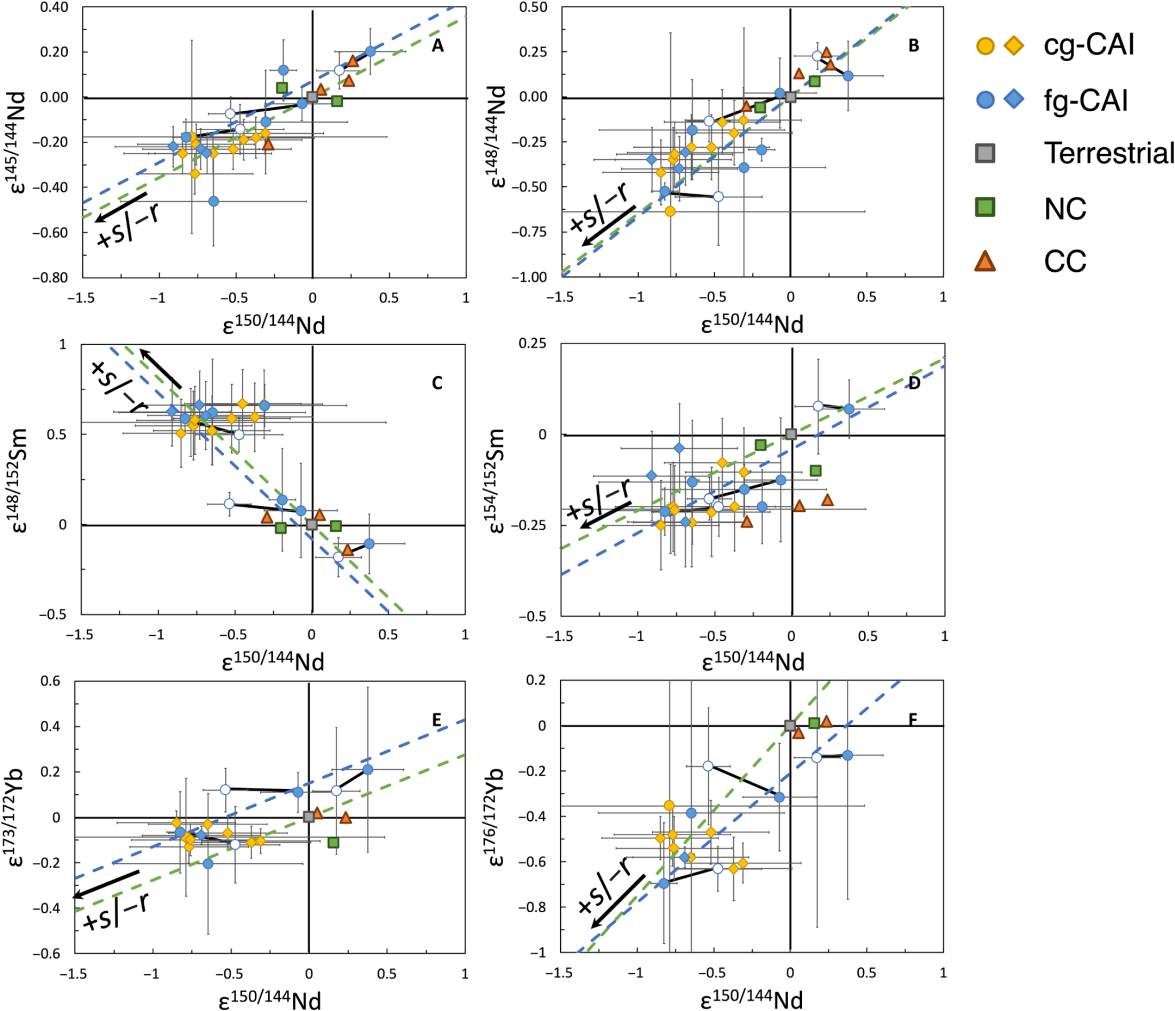
Cross-correlation diagrams showing variations in the contributions of *r*- and *s*-isotopes in CAIs and chondrites. (**A** to **F**) Plots of (A) ε^145/144^Nd, (B) ε^148/144^Nd, (C) ε^148/152^Sm, (D) ε^154/152^Sm, (E) ε^173/172^Yb, and (F) ε^176/172^Yb against ε^150/144^Nd. Variations in the *p*-process contribution would not be visible in these diagrams as no *p*-process isotope is used in defining any of the ratios plotted here. Circles are CAIs analyzed in this study, and the open ones replicate analyses of *FGft*-4, *FGft*-8, and *FGft*-9. Diamonds are from (*23*). NC and CC compositions are from a compilation in (*5*). See the description of error bar calculations in Materials and Methods. The blue dashed lines are the best-fit lines for fg-CAIs using IsoplotR. The green dashed lines are modeled mixing lines (*32*) between a terrestrial component (gray cubes) and an end-member with *r*/*s*-deficits or excesses originating from a low-mass AGB star (*28*).

Nucleosynthetic anomalies of Zr, Mo, and Ru have previously been observed in CAIs, which appear to be most consistent with either *r*-excesses (*12*, *13*) or *s*-deficits (*14*, *37*). Multiple isotopes of Nd, Sm, and Yb with primarily *r*/*s*-process origins in the fg-CAIs show correlations that follow predicted lines of *r*-deficits or *s*-excesses inherited from low-mass AGB stars (Fig. 5) (*28*), with most fg-CAIs on the *s*-excess or *r*-deficit side but spanning a significant range covering the average compositions of the NC clan (terrestrial, H, L, LL, EH, and EL) and CC clan (CI, CM, CO, CV, and CR) bulk materials. It is worth noting that the documented isotopic variations in fg-CAIs cannot be due to contamination from the matrix during CAI extraction because the LREEs, such as Nd and Sm, are typically >10 times more concentrated in fg-CAIs compared to matrix material. It would therefore take a volume of matrix several tens of times greater than that of the fg-CAIs to cause such isotopic variations.

### Correlations of anomalies of ^50^Ti and *p*-process isotopes in CAIs

**Fig. 6.**
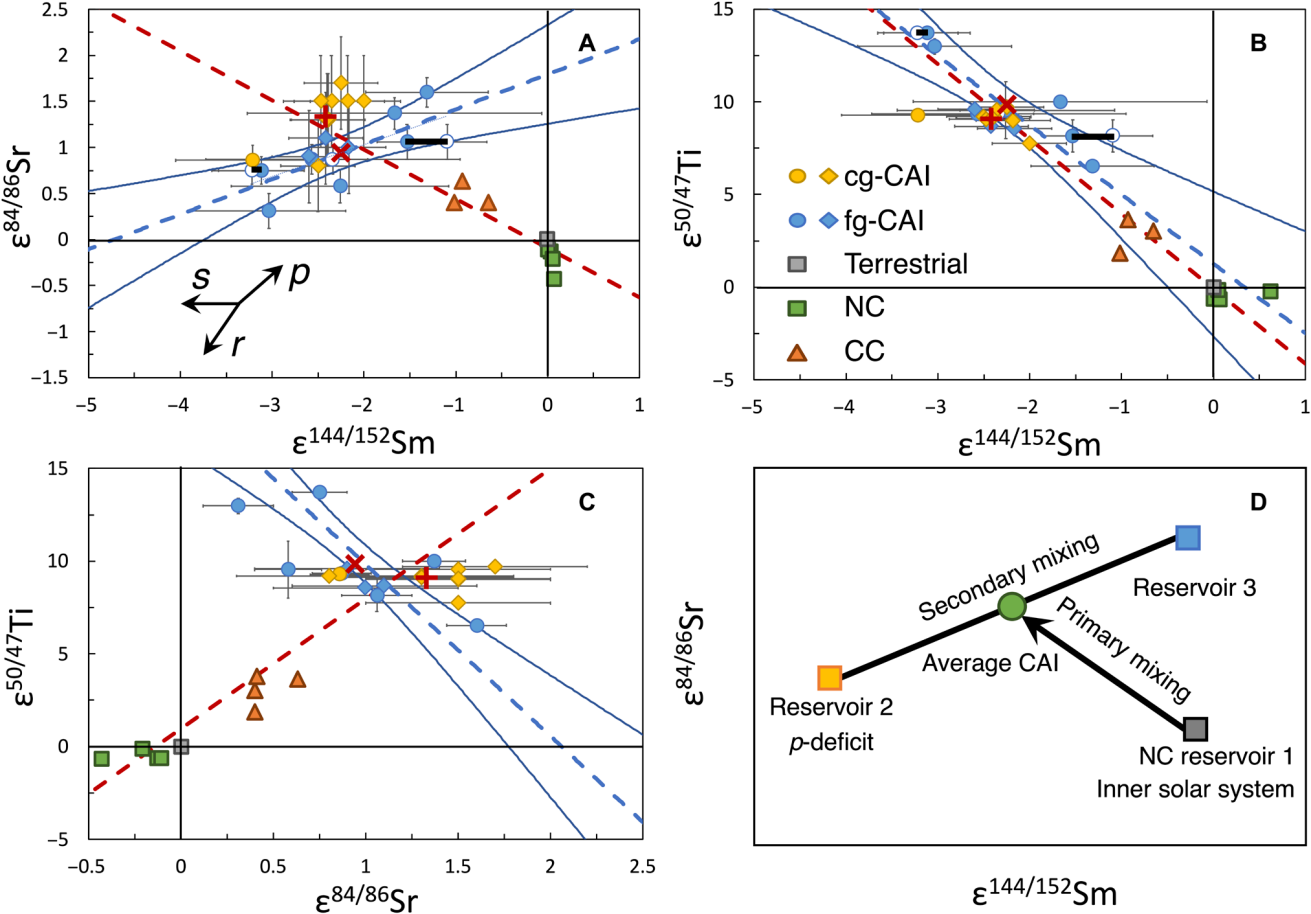
Cross-correlation diagrams of isotopic anomalies of ^50^Ti and *p*-isotopes in CAIs. (**A** to **D**) Plots of ε^84/86^Sr versus ε^144/152^Sm (A), ε^50/47^Ti versus ε^144/152^Sm (B), ε^50/47^Ti versus ε^84/86^Sr (C), and a cartoon for mixing (D). Note that isotopic variations in ε^144/152^Sm and ε^84/86^Sr can also arise from variations in *s*- and *r*-isotopes as these are involved in the definition of ε-values through internal normalization. Circles represent CAIs analyzed in this study and (*29*, *36*), with the open circles representing replicates of *FGft*-4, *FGft*-8, and *FGft*-9. Diamonds are from (*23*). NC and CC compositions are from a compilation in (*5*). The red plus and cross symbols are the average of cg- and fg-CAIs, respectively. The linear regressions (blue dashed lines) and 2σ envelopes (blue lines) are calculated for fg-CAIs using IsoplotR and York’s linear regression (*48*), respectively. The linear regressions of NC, CCs, and CAI averages are plotted as red dashed lines. Plots involving the *p*-process isotope ^164^Er show no correlation (Supplementary Materials), possibly due to the very large uncertainty of ε^164/168^Er analyses.

Figure 5 shows that some of the isotopic variations documented for REEs in CAIs are controlled by varying contributions of the *s*- and *r*-processes of nucleosynthesis. As discussed in the “Confirming the presence of *p*-isotope anomaly in ε^144/152^Sm” section, for elements like Sm, variations in the *p*-process also played a significant role. This is further illustrated in Fig. 6 where we plot isotopic variations involving *p*-process isotopes [^84^Sr, (*36*); ^144^Sm, this study and (*23*)] as well as isotopic anomalies in ^50^Ti (*29*). A correlation is also found between ε^50/47^Ti and ε^144/152^Sm that extends to bulk NC and CC meteorites (Fig. 6B), consistent with the view that for those elements, the CC reservoir’s isotopic compositions can be explained as a mixture between NC and material with isotopic compositions similar to those of refractory inclusions but a chemical composition that is closer to solar composition [inclusion-like or IC component, as described in (*9*)].

The cross-correlation diagram ε^84/86^Sr-ε^144/152^Sm reveals more complexity. To a first order, we find again that CC lies between NC and the average composition of CAIs (IC component). However, many fg-CAIs do not plot on this correlation and define instead a secondary correlation. These variations therefore require mixing between three reservoirs. In a ε^84/86^Sr-ε^144/152^Sm diagram, ternary mixing would produce scatter bound by binary mixing lines. What we see instead are two distinct correlations, which is more consistent with two binary mixing events between nucleosynthetically distinct isotopic reservoirs. The presence of a similar secondary mixing line had previously been observed for ε^50/47^Ti versus ε^84/86^Sr in the same fg-CAIs (*36*), which is plotted in Fig. 6C.

The primary correlation between NCs-CCs-average CAIs (Fig. 6D) cannot be explained by a single process, although other REE isotopes point to *s*-process excess (Fig. 1) (*5*, *9*), but this would need to be combined with a *p*-excess to explain the data. We favor a *p*-origin for the secondary correlations among fg-CAIs (Fig. 6D) because (i) *p*-origin is an excellent match to the observed secondary slope, bearing in mind that this slope can also be affected by elemental fractionation (*32*); (ii) all Sm isotopes together seem to implicate the *p*-process (Fig. 4) (*5*); and (iii) leachates of fg-CAIs have revealed the presence of large ^84^Sr anomalies in these objects (*38*).

### Transport of fg-CAIs in the infant SS

Measured REE mass–dependent isotopic data for the fg-CAIs with group II REE patterns suggest that they have been subject to intense heating that is possibly related to the FUor or Exor outbursts, where the fg-CAI precursors may have experienced cycles of evaporation and condensation near the Sun in multiple outbursts (*22*). During an outburst of FUor events, the dusty envelope above the disk could be displaced by a strong wind, which could have transported material out as far as 10 AU until it was resisted by the envelope (*39*). In this picture, CAI precursors have been transported by wind (*40*) to the wind-envelope interaction zone (WEIZ) (*39*), where the precursors were contaminated by the dust of outer SS material.

CAIs, with their high-temperature, volatile-depleted chemical compositions, are expected to be primarily derived from inner SS material. The carriers of isotopic anomalies likely have extreme isotopic anomalies and would therefore constitute only a small fraction of the CAIs. Such objects are not uncommon in the SS and include presolar grains, FUN CAIs, and hibonite grains. For instance, hibonite grains have ε^50/47^Ti isotopic anomalies ranging from −750 to +1000 (*41*, *42*). The incorporation of just ~1% of material like hibonite grains could account for the Ti isotopic anomalies observed in fg-CAIs.

The dust in the envelope could potentially be chemically and isotopically distinct from the average SS composition, as it has not yet been homogenized in the disk. As CAI precursors are transported by the wind to the WEIZ, they may continue to grow through collisions with each other and a small fraction of outer SS material carrying extreme Ti and *p*-isotopic anomalies. However, collisions are generally inefficient at mixing compact grains, potentially taking about 20 times longer to homogenize the material than to grow it (*43*). These large CAIs, once they fall back to the disk, could also increase the local dust–to–gas ratio, potentially triggering instabilities that accelerate accretion (*44*).

**Fig. 7.**
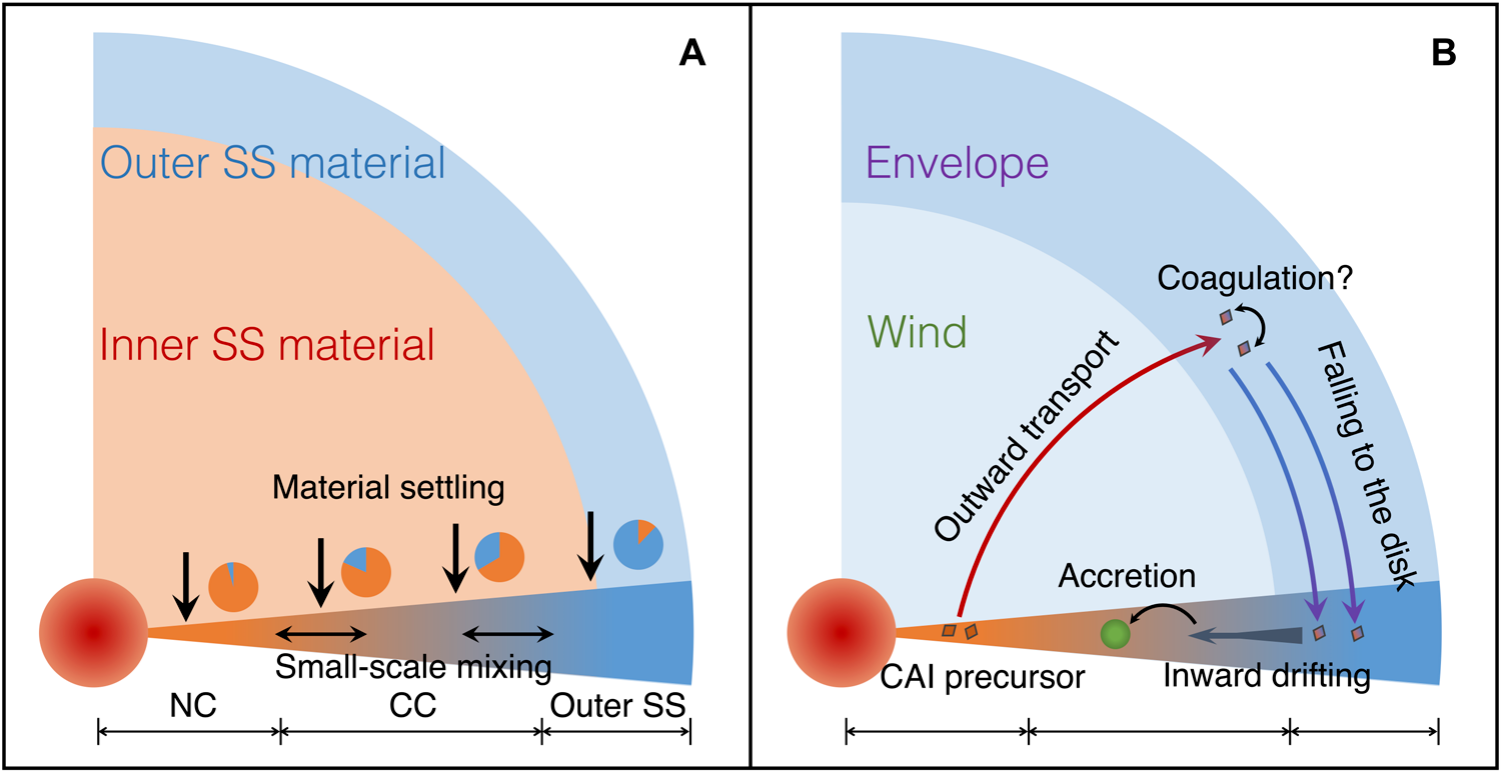
Schematics of the protoplanetary disk before and during CAI formation and transport. CAI precursors are formed in (**A**) and transported to outer disk in (**B**). The figure is modified from figure 9 in (*39*).

In this context, we propose the following scenarios to explain the nucleosynthetic anomalies in normal CAIs (Fig. 7):

1) The inner SS was characterized by an isotopic composition most similar to NC. The inner SS material was in the central region of the cloud core, while the outer SS material was distributed in the external region. As the nebula collapsed, the inner SS material settled early, preferentially into the inner disk and the protosun. The outer SS material, starting further away and with more angular momentum, reached the disk later (Fig. 7A).

2) After the disk formed, the inner disk material experienced intense, cyclic heating of FUor or Exor outbursts. CAI precursors were launched from the disk in solid form by the wind driven by the outburst and transported to the outer SS region as the envelope was pushed back (*39*). The CAI precursors carried by the wind traveled to the outer SS region, possibly beyond the accretion region of the CC clan. Within the WEIZ, the precursors aggregated as they collided with each other and outer SS material (Fig. 7B). The outer SS envelope was characterized by a uniform *s*-excess, with variable contributions of the *p*-process.

3) CAIs large enough fell out of the WEIZ, while smaller ones stayed in the WEIZ until the wind subsided and the envelope fell back onto the disk. The CAIs would then have drifted inward toward the Sun. Most were captured in the accretion region of the CC clan (*45*). During drifting, CAIs of different sizes may have been dynamically decoupled and ended up in CCs of different types. The formation of Jupiter may have prevented most CAIs from drifting further inward and being accreted into NC material (Fig. 7B).

To summarize, in fine-grained refractory inclusions, we find evidence for secondary mixing lines involving *p*-process isotopes ^84^Sr and ^144^Sm and neutron-rich isotope ^50^Ti. One possible interpretation of these variations is that CAI precursors started with NC compositions, but they experienced mixing with material enriched in the *s*-processes and marked by variable *p*-process excesses when they were launched outward by winds from the young active sun, resulting in contamination of these inclusions with envelope material. The manner in which such contamination could have taken place is uncertain but could have involved coagulation resulting from van der Waals forces (*43*).

## MATERIALS AND METHODS

### Sample processing

The isotopic anomalies of the REEs in the CAIs were analyzed along with the MDFs previously reported in (*22*). Sample selection and digestion, REE extraction and purification, and MC-ICPMS (multicollector inductively coupled plasma mass spectrometer) analysis have been described in detail in (*22*) and are only briefly summarized below.

The samples used were selected from a set of well-characterized fg-CAIs with group II REE patterns that were previously studied in (*46*). The fg-CAIs with masses ranging from 15 to 440 mg were extracted from a few Allende slabs using stainless-steel dental tools. One cg-CAI (TS32) was also digested along with the fg-CAIs for comparison. All CAIs were ground to fine powder and digested in HF/HNO_3_ in a 3:1 proportion with a few drops of HClO_4_ added. The samples in acid were then placed on a hot plate and heated to 160°C for 2 weeks before being dried down and redissolved twice in a 2:1 mixture of HCl:HNO_3_ to remove fluorides. After removing the fluoride, the samples were dried down, dissolved in a few drops of concentrated HNO_3_, and then diluted in 3 M HNO_3_ and centrifuged.

Approximately 80% of the digested samples were taken and passed through two UTEVA columns for U extraction. The matrix cuts that contained all the REEs were collected after UTEVA chemistry. Approximately 30% of the matrix cuts (equivalent to ~24% fraction of the whole CAI) were then processed through a prepacked *N*,*N*,*N*′,*N*′-tetraoctyl diglycolamide (TODGA) column to extract the bulk REEs with a yield near 100%. Three fg-CAIs (*FGft-*4, *FGft-*8, and *FGft-*9) were also analyzed using another 40% of matrix cuts of the UTEVA chemistry as replicates. These samples have been passed through a separate Mo chemistry, during which 40 to 90% of LREEs and 20 to 40% of HREEs (heavy REEs) were lost before the REEs were extracted. These three duplicates were only used to assess reproducibility for the MDF measurements in (*22*) and gave generally consistent results despite the loss of the REEs on the column. In this study of isotopic anomalies, these replicates are similarly used to assess the reproducibility of the measurements.

The extracted bulk REEs were first subjected to fluoropolymer-pneumatic liquid chromatography (FPLC) elutions for the general separation of the REEs (*22*, *47*). After the initial elution, REEs not neighboring each other were recombined and loaded for a second FPLC elution step to remove isobaric interferences and matrices. Readers are referred to (*22*) for a detailed description of FPLC elution. The total procedure blanks (<0.25 ng for all REEs) were more than two orders of magnitude lower than the sample amount for all the analyzed REEs.

### Isotopic analyses

The isotopic compositions of Nd, Sm, Gd, Dy, Er, and Yb were analyzed at the University of Chicago on a Thermo Fisher Scientific MC-ICPMS upgraded to Neptune Plus specifications. The purified REEs were introduced using an Apex Omega desolvating nebulizer. Data reduction was performed by copying the raw data into a spreadsheet to correct for background and isobaric interferences.

For data reduction, the isotopic ratios for all cycles were averaged. Because MDF is pervasive in CAIs, the isotope ratio of each measurement was internally normalized, assuming an exponential mass fractionation law to eliminate MDF. The isotopic ratios after internal normalization in the sample were then bracketed to those of standards to calculate epsilon values

εi/jE=10,000[2(Ei/Ej)SMP(Ei/Ej)STD,1+(Ei/Ej)STD,2−1] (1)

where (Ei/Ej)SMP is the isotope ratio of the sample, and (Ei/Ej)STD,1 and (Ei/Ej)STD,2 are the isotopic ratios of the standards analyzed before and after the sample, respectively.

LREEs were measured approximately nine times at concentrations of 15 to 25 parts per billion (ppb), corresponding to signals of 3.5 to 10 V for the most abundant isotopes (4 V for ^142^Nd and 3.5 V for ^152^Sm). Eu and HREEs were depleted in the CAIs and measured one to six times at 1.5 to 10 ppb, producing signals of 1.5 to 3 V. An exception is Er in *FGft*-3, *FGft*-6, *FGft*-7, and *FGft*-10, where extreme depletion at 1.5 ppb yielded a 0.25-V signal for ^166^Er.

The $\varepsilon^{i/j}E$ values obtained for the same sample were averaged and are reported in tables S1 and S2. The uncertainty is given as 95% confidence intervals using Student’s *t* value and either the variability of the sample $\varepsilon^{i/j}E$ values for those with six or more values or the variability of the $\varepsilon^{i/j}E$ values of standards bracketed by standards for sample measurements with less than six values.

The influence of *r*/*s*-deficits or excesses was evaluated by comparing the measured values to modeled mixing lines that pass a terrestrial component and an end-member with *r*/*s*-deficits or excesses that originated from a low-mass AGB star (Figs. 1, 4, and 5). The modeled mixing lines are calculated following equation B.5 in (*33*), with the composition of *r*/*s*-process contribution adopted from (*28*). The best-fit lines in Figs. 5 and 6 are calculated using IsoplotR, and the 2σ envelopes in Fig. 6 are calculated using York *et al.*’s approach (*48*).

## Supplementary Materials

### This PDF file includes:

Supplementary Text Figs. S1 to S4 Tables S1 to S3 References
