# Supplementary material for: Rare earth element nucleosynthetic anomalies and dust transport in the protoplanetary disk
Source: Sci Adv. 2025 Jul 9;11(28):eadv3148. doi: 10.1126/sciadv.adv3148 (PMC12239934; doi:10.1126/sciadv.adv3148)
Supplement: Supplementary file 1 — Supplementary Text Figs. S1 to S4 Tables S1 to S3 References [file sciadv.adv3148_sm.pdf]

Science Advances
AAAS

Supplementary Materials for

Rare earth element nucleosynthetic anomalies and dust transport in the protoplanetary disk

Justin Y. Hu *et al.*

Corresponding author: Justin Y. Hu, jh2363@cam.ac.uk

Sci. Adv. **11**, eadv3148 (2025)
DOI: 10.1126/sciadv.adv3148

This PDF file includes:

Supplementary Text
Figs. S1 to S4
Tables S1 to S3
References

Supplementary Text

1. Contributions of different sources calculated from isotope anomalies.

Assuming that the target element has m isotopes contributed from n different sources ($m > n$). The fraction of source j in the sample is x_j,

$\sum_{j=1}^{n} x_j = 1.$ (B1)

The fraction of isotope i in the source j is $f_{i,j}$,

$\sum_{i=1}^{m} f_{i,j} = 1.$ (B2)

In our case involving Sm, which has $m = 7$ isotopes, the $f_{i,j}$ values would correspond to the fraction of each isotope of an element contributed by the $n = 3$, (*p*-, *s*-, and *r*-processes). In that picture, the terrestrial composition is obtained by weighing evenly each component and multiplying by the absolute terrestrial isotopic abundances N_i conventionally normalized to 10^6 atoms (*50*), $\sum_{j=1}^{n} f_{i,j} N_i$.

If each source is weighted equally, the sample will show no isotope anomalies. Otherwise, the isotope anomalies in the sample for isotope i, ε_i, follows relationship,

$$\varepsilon_i = \left(\frac{\sum_{j=1}^{n} x_j f_{i,j}}{\sum_{j=1}^{n} \frac{1}{n} f_{i,j}} - 1\right) 10^4. \qquad \text{(B3)}$$

Simplifying Eq. B3 with Eq. B2, one has,

$$\sum_{j=1}^{n} x_j f_{i,j} = \frac{1}{n}\left(1 + \frac{\varepsilon_1}{10^4}\right). \qquad \text{(B4)}$$

We therefore can write Eq. B4 in the matrix form,

$$\begin{pmatrix} f_{1,1} & f_{1,2} & \cdots & f_{1,n} \\ f_{2,1} & f_{2,2} & \cdots & f_{2,n} \\ & \cdots & & \\ f_{m,1} & f_{m,2} & \cdots & f_{m,n} \end{pmatrix} \begin{pmatrix} x_1 \\ x_2 \\ \cdots \\ x_n \end{pmatrix} = \frac{1}{n} \begin{pmatrix} 1 + \frac{\varepsilon_1}{10^4} \\ 1 + \frac{\varepsilon_2}{10^4} \\ \cdots \\ 1 + \frac{\varepsilon_m}{10^4} \end{pmatrix}. \qquad \text{(B5)}$$

Solving Eq. B5, one gets the contributions from different sources,

$$\begin{pmatrix} x_1 \\ x_2 \\ \cdots \\ x_n \end{pmatrix} = \frac{1}{n} \begin{pmatrix} f_{1,1} & f_{1,2} & \cdots & f_{1,n} \\ f_{2,1} & f_{2,2} & \cdots & f_{2,n} \\ & \cdots & & \\ f_{m,1} & f_{m,2} & \cdots & f_{m,n} \end{pmatrix}^{-1} \begin{pmatrix} 1 + \frac{\varepsilon_1}{10^4} \\ 1 + \frac{\varepsilon_2}{10^4} \\ \cdots \\ 1 + \frac{\varepsilon_m}{10^4} \end{pmatrix}. \qquad \text{(B6)}$$

To calculate the contributions of *p*-, *s*-, and *r*-process materials, the target element needs to have at least 3 isotopes for decomposition. Samarium has 5 isotopes (^{144}Sm, ^{147}Sm, ^{148}Sm, ^{152}Sm, and ^{154}Sm) excluding ^{149}Sm and ^{150}Sm, which are affected by cosmic-ray irradiation. The contributions from *p*-, *s*-, and *r*-process materials in the SS are obtained from (*28*). One can therefore calculate the contributions from *p*-, *s*-, and *r*-process materials for the CAIs.

2. Correlation of radiogenic Nd isotopes in the fg-CAIs

$^{143}Nd/^{144}Nd$ is correlated to $^{147}Sm/^{144}Nd$ with a slope corresponding to an approximate model age of 3.987 Gy (fig. S4A). No obvious correlation is observed between $\varepsilon^{142/144}Nd$ and $\varepsilon^{143/144}Nd$ due to larger uncertainties in $\varepsilon^{142/144}Nd$ (fig. S4B). The model age of the CAIs in (*23*) is 4.548 Gy. The lower-than-expected model age in the fg-CAIs is likely a combination of random and systematic errors in $^{147}Sm/^{144}Nd$ ratio determination, as this was not the main scope of this work. For reference, we have calculated the $^{147}Sm/^{144}Nd$ in each CAI based on their $\varepsilon^{143/144}Nd$ values and an assumed age of 4.567 Ga and these are compiled in table S3.

3. Plots of isotope anomalies against MDF for Ti, Sr, Sm, and Er.

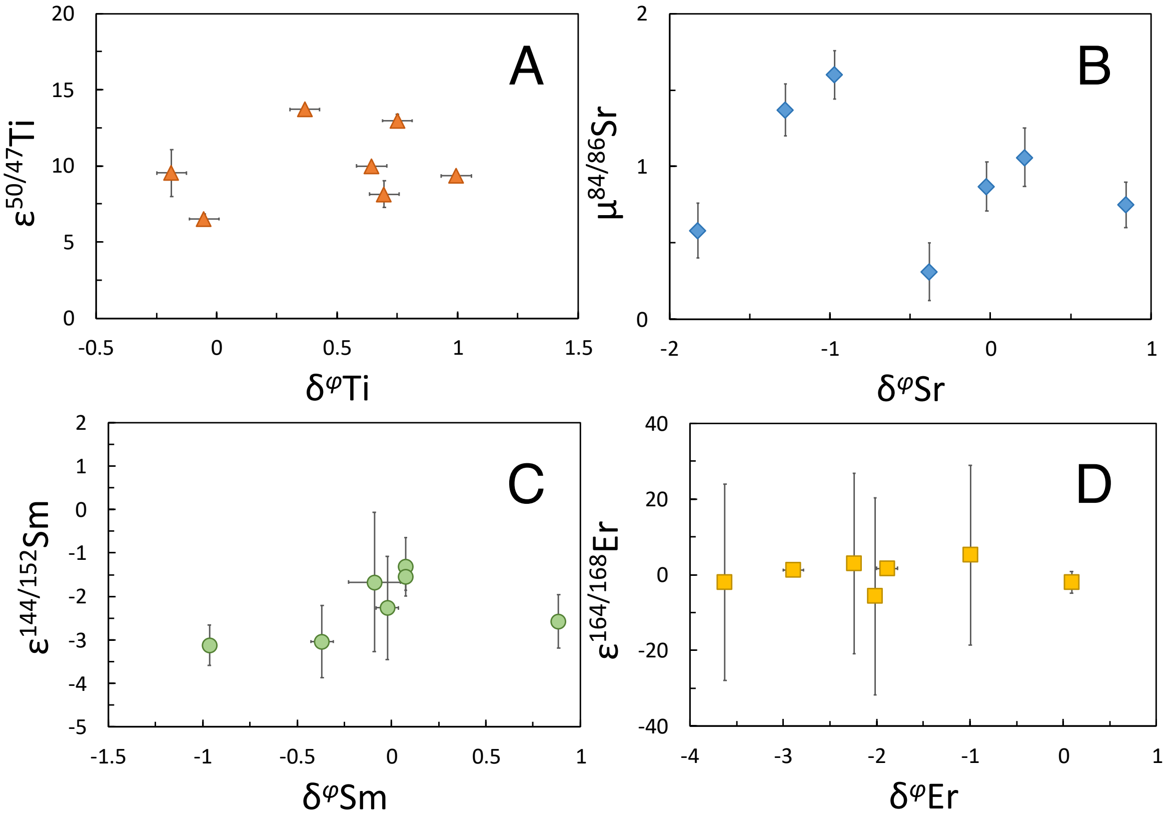

Fig. S1 Plots of isotope anomalies against MDFs for Ti (A), Sr (B), Sm (C), and Er (D). Mass dependent fractionations are present in ‰/amu.

4. Lack of correlations between r/s-isotopes and ^{50}Ti/p-isotopes in the CAIs

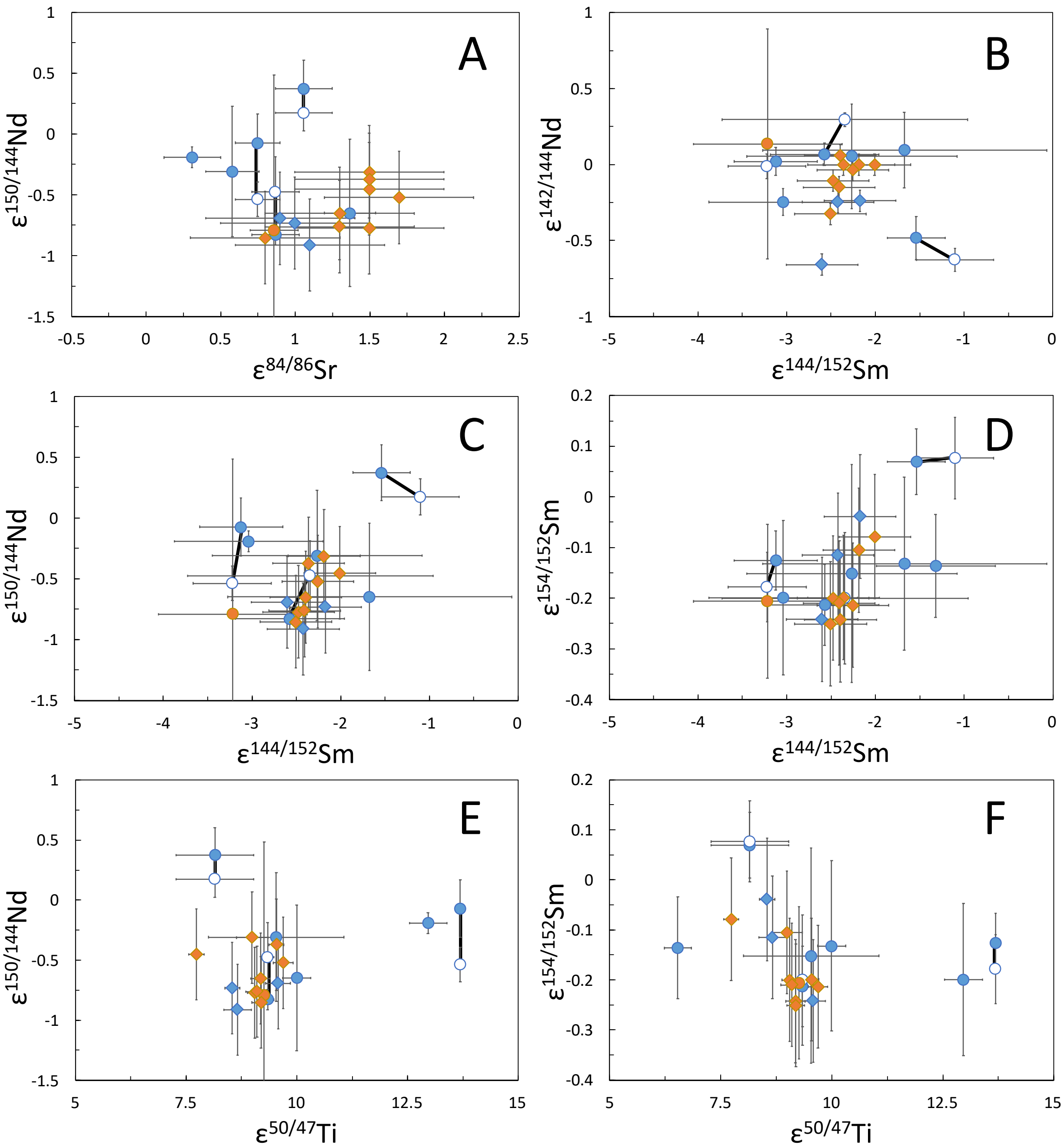

Fig. S2 Cross correlation diagrams between *r*/*s*-isotopes and ^{50}Ti and *p*-isotopes in the CAIs. Panels **A-F** show plots for (**A**) $\varepsilon^{150/144}$Nd *vs*. $\varepsilon^{84/86}$Sr, (**B**) $\varepsilon^{142/144}$Nd *vs*. $\varepsilon^{144/152}$Sm, (**C**) $\varepsilon^{150/144}$Nd *vs*. $\varepsilon^{144/152}$Sm, (**D**) $\varepsilon^{154/152}$Sm *vs*. $\varepsilon^{144/152}$Sm, (**E**) $\varepsilon^{150/144}$Nd *vs*. $\varepsilon^{50/47}$Ti, (**F**) $\varepsilon^{154/152}$Sm *vs*. $\varepsilon^{50/47}$Ti, respectively. Blue dots are for fg-CAIs and orange dots are for cg-CAIs. Circles correspond to CAIs analyzed in this study and (*29*, *36*), with the open symbols being replicates (*FGft*-4, -8, -9). Diamonds are from (*23*).

5. Isotopic anomalies of ^{164}Er plotted against ^{50}Ti and ^{84}Sr in CAIs.

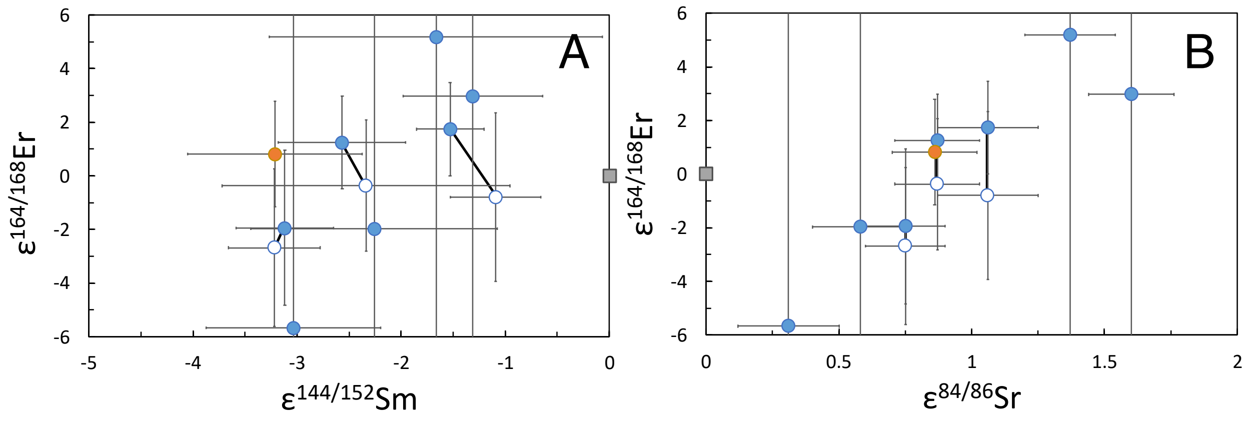

Fig. S3 Isotopic anomalies of ^{164}Er plotted against ^{50}Ti (A) and ^{84}Sr (B) in CAIs. Note that isotopic variations in $\varepsilon^{164/168}$Er and $\varepsilon^{84/86}$Sr can also arise from variations in *s*- and *r*-isotopes as these are involved in the definition of ε-values through internal normalization. Circles represent CAIs analyzed in this study and (*36*), with the open circles representing replicates of *FGft*-4, -8, and -9. Blue and orange symbols are for fg- and cg-CAIs respectively. Gray cubes are terrestrial compositions.

6. Plot of ^{147}Sm-^{143}Nd isochrons and $\varepsilon^{143/144}Nd$ *vs.* $\varepsilon^{142/144}Nd$ for the CAIs.

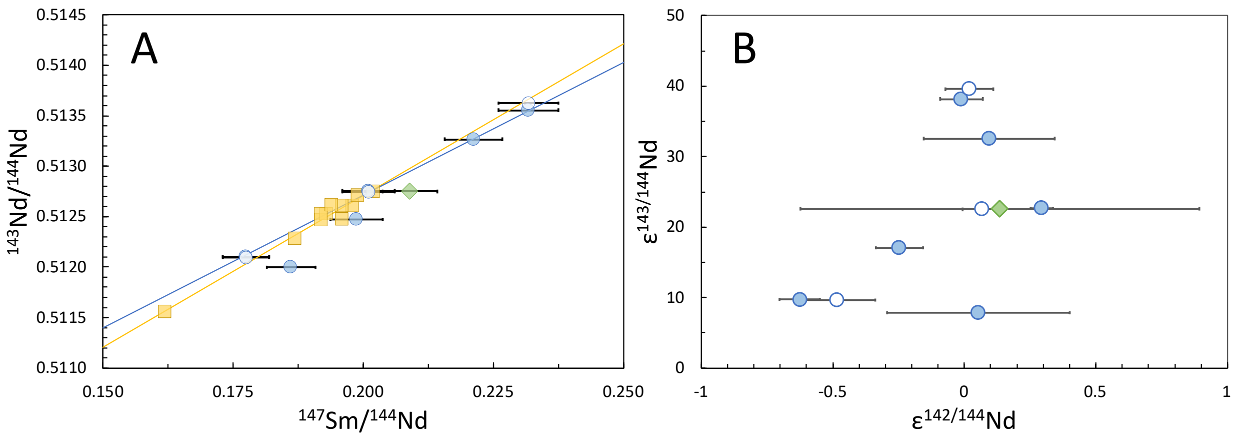

Fig. S4 Plot of ^{147}Sm-^{143}Nd isochrons (A) and $\varepsilon^{143/144}Nd$ *vs.* $\varepsilon^{142/144}Nd$ (B) for the CAIs. The yellow squares and yellow lines are CAI measurements and the isochron from (*23*). The diamond and circles are from the CAI measurements in this study (table S1 for isotopes). The Sm/Nd ratio was measured using laser ablation ICP-MS. The green diamond is for the cg-CAI TS32. The blue circles are for the fg-CAIs, with the outline ones being the replicates. The blue line represents the isochron calculated exclusively from the fg-CAIs, with replicates averaged. The random errors on $^{147}Sm/^{144}Nd$ are assumed to be 2.5% but the systematic error could be larger.

7. Internally-normalized isotopic compositions of Nd, Sm, and Gd.

Table S1 Internally-normalized isotopic compositions of Nd, Sm, and Gd in the geostandard BCR-2 and CAIs.

Sample name	Nick name	^{142}Nd/^{144}Nd	^{143}Nd/^{144}Nd	^{145}Nd/^{144}Nd	^{148}Nd/^{144}Nd	^{150}Nd/^{144}Nd	#
BCR-2		0.25 ± 0.09	20.15 ± 0.07	-0.03 ± 0.05	-0.03 ± 0.11	0.08 ± 0.19	15
TS32		0.14 ± 0.76	22.61 ± 0.71	-0.18 ± 0.43	-0.64 ± 0.99	-0.79 ± 1.27	4
ME-3364-25.2	*FGft-3*	0.09 ± 0.25	32.55 ± 0.23	-0.46 ± 0.20	-0.18 ± 0.28	-0.65 ± 0.61	9
ME-2639-16.2	*FGft-4*	0.30 ± 0.04	22.69 ± 0.09	-0.14 ± 0.11	-0.56 ± 0.27	-0.47 ± 0.29	8
ME-2639-16.2	*FGft-4**	0.07 ± 0.07	22.52 ± 0.07	-0.18 ± 0.08	-0.53 ± 0.05	-0.83 ± 0.09	8
ME-2639-49.7	*FGft-6*	-0.25 ± 0.09	17.07 ± 0.10	0.12 ± 0.14	-0.29 ± 0.06	-0.19 ± 0.09	5
AL3S5	*FGft-8*	-0.62 ± 0.08	9.74 ± 0.07	0.12 ± 0.08	0.23 ± 0.07	0.17 ± 0.15	8
AL3S5	*FGft-8**	-0.48 ± 0.14	9.68 ± 0.15	0.20 ± 0.10	0.12 ± 0.19	0.38 ± 0.23	15
AL4S6	*FGft-9*	-0.01 ± 0.08	38.17 ± 0.05	-0.07 ± 0.08	-0.14 ± 0.11	-0.54 ± 0.14	11
AL4S6	*FGft-9**	0.02 ± 0.09	39.55 ± 0.09	-0.03 ± 0.07	0.02 ± 0.19	-0.07 ± 0.24	13
AL8S2	*FGft-10*	0.05 ± 0.35	7.85 ± 0.18	-0.11 ± 0.23	-0.39 ± 0.78	-0.31 ± 0.54	3
**Sample name**	**Nick name**	**^{144}Sm/^{152}Sm**	**^{148}Sm/^{152}Sm**	**^{149}Sm/^{152}Sm**	**^{150}Sm/^{152}Sm**	**^{154}Sm/^{152}Sm**	**#**
BCR-2		-0.16 ± 0.46	0.01 ± 0.13	0.10 ± 0.04	-0.02 ± 0.14	0.08 ± 0.06	9
TS32		-3.21 ± 1.63	0.57 ± 0.26	-0.45 ± 0.22	1.40 ± 0.70	-0.21 ± 0.20	2
ME-3364-25.2	*FGft-3*	-1.67 ± 1.60	0.62 ± 0.26	-0.57 ± 0.29	1.99 ± 0.43	-0.13 ± 0.17	5
ME-2639-16.2	*FGft-4*	-2.57 ± 0.61	0.59 ± 0.17	-0.33 ± 0.12	1.32 ± 0.12	-0.21 ± 0.08	3
ME-2639-16.2	*FGft-4**	-2.34 ± 1.38	0.50 ± 0.11	-0.42 ± 0.08	1.22 ± 0.17	-0.20 ± 0.13	5
ME-2639-49.7	*FGft-6*	-3.04 ± 1.30	0.14 ± 0.29	-0.42 ± 0.27	0.99 ± 0.37	-0.20 ± 0.17	2
ME-2639-51.1	*FGft-7*	-1.31 ± 0.67	0.47 ± 0.28	-0.48 ± 0.20	1.55 ± 0.38	-0.14 ± 0.10	5
AL3S5	*FGft-8*	-1.53 ± 0.32	-0.11 ± 0.07	-0.37 ± 0.07	0.89 ± 0.07	0.07 ± 0.07	9
AL3S5	*FGft-8**	-1.10 ± 0.44	-0.18 ± 0.09	-0.45 ± 0.08	0.79 ± 0.12	0.08 ± 0.08	11
AL4S6	*FGft-9*	-3.12 ± 0.47	0.08 ± 0.07	-0.32 ± 0.08	1.19 ± 0.12	-0.13 ± 0.06	9
AL4S6	*FGft-9**	-3.22 ± 0.44	0.11 ± 0.12	-0.42 ± 0.09	1.20 ± 0.07	-0.18 ± 0.07	7
AL8S2	*FGft-10*	-2.26 ± 1.30	0.66 ± 0.29	-0.43 ± 0.27	1.38 ± 0.37	-0.15 ± 0.17	1
**Sample name**	**Nick name**			**^{155}Gd/^{156}Gd**	**^{157}Gd/^{156}Gd**	**^{158}Gd/^{156}Gd**	**#**
BCR-2				0.01 ± 0.06	0.02 ± 0.11	-0.01 ± 0.08	10
TS32				-0.12 ± 0.37	-0.39 ± 0.28	0.39 ± 0.25	2
ME-3364-25.2	*FGft-3*			0.03 ± 0.37	-0.12 ± 0.28	0.40 ± 0.25	1
ME-2639-16.2	*FGft-4*			-0.10 ± 0.18	-0.45 ± 0.20	0.25 ± 0.12	2
ME-2639-16.2	*FGft-4**			-0.15 ± 0.37	-0.38 ± 0.28	0.36 ± 0.25	6
ME-2639-49.7	*FGft-6*			0.01 ± 0.35	0.09 ± 0.17	0.22 ± 0.12	3
ME-2639-51.1	*FGft-7*			-0.31 ± 0.15	-0.59 ± 0.16	0.39 ± 0.31	3
AL3S5	*FGft-8*			-0.12 ± 0.14	-0.57 ± 0.09	0.32 ± 0.05	9
AL3S5	*FGft-8**			-0.01 ± 0.15	-0.62 ± 0.15	0.40 ± 0.10	10
AL4S6	*FGft-9*			-0.27 ± 0.22	-0.61 ± 0.05	0.24 ± 0.16	5
AL4S6	*FGft-9**			-0.07 ± 0.36	-0.17 ± 0.20	0.28 ± 0.13	4
AL8S2	*FGft-10*			-0.26 ± 0.33	0.33 ± 0.20	0.37 ± 0.34	2

1 Expressed in epsilon units $\varepsilon^{i/j}E = 10000[\,({}^{i}E/{}^{j}E)_{smp}/({}^{i}E/{}^{j}E)_{std}-1]$ after internal normalization of the isotopic ratios (^{146}Nd/^{144}Nd = 0.7219; ^{147}Sm/^{152}Sm = 0.56081; (^{155}Gd + ^{156}Gd)/^{160}Gd = 1.6129)

2 BCR-2 is a terrestrial geostandard, TS32 is a cg-CAI, and the rest are fg-CAIs

Number of measurements

* Replicates after REE extraction

8. Internally-normalized isotopic compositions of Dy, Er, and Yb

Table S2 Internally-normalized isotopic compositions of Dy, Er, and Yb in the geostandard BCR-2 and CAIs.

Sample name	Nick name			$^{161}Dy/^{162}Dy$	$^{163}Dy/^{162}Dy$	#
BCR-2				0.05 ± 0.12	0.04 ± 0.06	8
TS32				0.19 ± 0.14	0.08 ± 0.23	3
ME-3364-25.2	*FGft-3*			-0.01 ± 0.30	-0.05 ± 0.23	1
ME-2639-16.2	*FGft-4*			0.09 ± 0.16	0.02 ± 0.06	2
ME-2639-16.2	*FGft-4**			0.13 ± 0.14	0.10 ± 0.09	6
ME-2639-49.7	*FGft-6*			0.50 ± 0.54	0.33 ± 0.15	3
ME-2639-51.1	*FGft-7*			0.02 ± 0.30	0.02 ± 0.23	2
AL3S5	*FGft-8*			0.08 ± 0.07	0.28 ± 0.09	9
AL3S5	*FGft-8**			0.14 ± 0.22	0.19 ± 0.14	8
AL4S6	*FGft-9*			0.10 ± 0.10	0.16 ± 0.08	7
AL4S6	*FGft-9**			0.18 ± 0.28	0.18 ± 0.21	5
AL8S2	*FGft-10*			-0.19 ± 0.48	0.42 ± 0.50	1

Sample name	Nick name		$^{164}Er/^{168}Er$	$^{167}Er/^{168}Er$	$^{170}Er/^{168}Er$	#
BCR-2			0.80 ± 1.95	-0.04 ± 0.13	0.13 ± 0.16	8
TS32			0.81 ± 2.98	-0.44 ± 0.45	0.20 ± 0.59	2
ME-3364-25.2	*FGft-3*		5.19 ± 25.25	0.83 ± 0.57	0.43 ± 3.61	1
ME-2639-16.2	*FGft-4*		1.25 ± 1.93	-0.08 ± 0.30	0.06 ± 0.53	1
ME-2639-16.2	*FGft-4**		-0.37 ± 1.93	0.00 ± 0.30	0.38 ± 0.53	2
ME-2639-49.7	*FGft-6*		-5.66 ± 24.43	0.01 ± 1.16	-0.38 ± 2.13	1
ME-2639-51.1	*FGft-7*		2.98 ± 25.25	0.22 ± 0.57	-0.01 ± 3.61	1
AL3S5	*FGft-8*		1.74 ± 1.93	-0.01 ± 0.30	0.33 ± 0.53	2
AL3S5	*FGft-8**		-0.79 ± 1.93	0.01 ± 0.30	0.06 ± 0.53	2
AL4S6	*FGft-9*		-1.94 ± 2.89	0.13 ± 0.10	0.67 ± 0.21	6
AL4S6	*FGft-9**		-2.68 ± 2.93	0.23 ± 0.29	0.72 ± 0.53	5
AL8S2	*FGft-10*		-1.97 ± 24.43	-0.55 ± 1.16	-0.08 ± 2.13	1

Sample name	Nick name	$^{170}Yb/^{172}Yb$	$^{171}Yb/^{172}Yb$	$^{173}Yb/^{172}Yb$	$^{176}Yb/^{172}Yb$	#
BCR-2		0.14 ± 0.88	0.24 ± 0.21	0.01 ± 0.12	0.00 ± 0.20	9
TS32		1.08 ± 3.37	0.09 ± 0.18	-0.09 ± 0.26	-0.35 ± 0.80	4
ME-3364-25.2	*FGft-3*	0.39 ± 7.00	-0.03 ± 0.84	-0.21 ± 0.31	-0.38 ± 1.63	3
ME-2639-16.2	*FGft-4*	1.32 ± 0.51	0.22 ± 0.18	-0.07 ± 0.18	-0.69 ± 0.27	4
ME-2639-16.2	*FGft-4**	0.39 ± 1.15	0.04 ± 0.34	-0.12 ± 0.17	-0.63 ± 0.10	9
ME-2639-51.1	*FGft-7*	0.84 ± 3.75	0.23 ± 0.49	0.33 ± 0.58	0.03 ± 0.85	1
AL3S5	*FGft-8*	7.50 ± 1.73	1.35 ± 0.40	0.21 ± 0.36	-0.13 ± 0.63	3
AL3S5	*FGft-8**	1.66 ± 1.05	1.66 ± 0.68	0.12 ± 0.28	-0.14 ± 0.75	2
AL4S6	*FGft-9*	0.43 ± 0.42	0.25 ± 0.10	0.11 ± 0.09	-0.31 ± 0.24	9
AL4S6	*FGft-9**	-0.37 ± 0.44	0.28 ± 0.17	0.12 ± 0.10	-0.18 ± 0.26	9

1 Expressed in epsilon units $\varepsilon^{i/j}E = 10000[(^iE/^jE)_{smp}/(^iE/^jE)_{std}-1]$ after internal normalization of the isotopic ratios ($^{164}Dy/^{162}Dy = 1.01237$; $^{166}Er/^{168}Er = 1.24140$; $^{174}Yb/^{172}Yb = 1.4772$)

2 BCR-2 is a terrestrial geostandard, TS32 is a cg-CAI, and the rest are fg-CAIs

Number of measurements

* Replicates after REE extraction

9. Calculated $^{147}Sm/^{144}Nd$ in each CAI.

Table S3 Calculated $^{147}Sm/^{144}Nd$ in each CAI based on their $\varepsilon^{143/144}Nd$ values and an assumed age of 4.567 Ga.

Sample	$^{143}Nd/^{144}Nd$	Measured $^{147}Sm/^{144}Nd$	Calculated $^{147}Sm/^{144}Nd$
TS32	0.512752	0.209	0.201
FG-FT-3	0.513266	0.221	0.219
FG-FT-4	0.512756	0.201	0.202
FG-FT-4*	0.512748	0.201	0.201
FG-FT-6	0.512472	0.199	0.192
FG-FT-8	0.512104	0.178	0.180
FG-FT-8*	0.512099	0.177	0.180
FG-FT-9	0.513555	0.232	0.228
FG-FT-9*	0.513628	0.232	0.231
FG-FT-10	0.511999	0.186	0.176

Calculated $^{147}Sm/^{144}Nd$ are obtained assuming the CAIs have a age of 4.548 Gy

*Replicates subjected to significatn loss of REEs during Mo chemistry and hence not used for data interpretation

REFERENCES AND NOTES

1. I. Pascucci, S. Cabrit, S. Edwards, U. Gorti, O. Gressel, T. Suzuki, The role of disk winds in the evolution and dispersal of protoplanetary disks. arXiv:2203.10068 (2022).

2. G. Lesur, B. Ercolano, M. Flock, M.-K. Lin, C.-C. Yang, J. Barranco, P. Benitez-Llambay, J. Goodman, A. Johansen, H. Klahr, G. Laibe, W. Lyra, P. Marcus, R.P. Nelson, J. Squire, J. B. Simon, N. Turner, O.M. Umurhan, A. N. Youdin, Hydro-, magnetohydro-, and dust-gas dynamics of protoplanetary disks. arXiv:2203.09821 (2022).

3. T. Birnstiel, Dust growth and evolution in protoplanetary disks. *Annu. Rev. Astron. Astrophys.* **62**, 157–202 (2024).

4. M. Audard, P. Abrahám, M. M. Dunham, J. D. Green, N. Grosso, K. Hamaguchi, J. H. Kastner, A. Kóspál, G. Lodato, M. Romanova, S. L. Skinner, E. I. Vorobyov, Z. Zhu, Episodic accretion in young stars. arXiv:1401.3368 (2014).

5. N. Dauphas, E. A. Schauble, Mass fractionation laws, mass-independent effects, and isotopic anomalies. *Annu. Rev. Earth Planet. Sci.* **44**, 709–783 (2016).

6. T. Kleine, G. Budde, C. Burkhardt, T. S. Kruijer, E. A. Worsham, A. Morbidelli, F. Nimmo, The non-carbonaceous–carbonaceous meteorite dichotomy. *Space Sci. Rev.* **216**, 55 (2020).

7. T. Hopp, N. Dauphas, Y. Abe, J. Aléon, C. M. O'D Alexander, S. Amari, Y. Amelin, K. Bajo, M. Bizzarro, A. Bouvier, R. W. Carlson, M. Chaussidon, B.-G. Choi, A. M. Davis, T. D. Rocco, W. Fujiya, R. Fukai, I. Gautam, M. K. Haba, Y. Hibiya, H. Hidaka, H. Homma, P. Hoppe, G. R. Huss, K. Ichida, T. Iizuka, T. R. Ireland, A. Ishikawa, M. Ito, S. Itoh, N. Kawasaki, N. T. Kita, K. Kitajima, T. Kleine, S. Komatani, A. N. Krot, M.-C. Liu, Y. Masuda, K. D. M. Keegan, M. Morita, K. Motomura, F. Moynier, I. Nakai, K. Nagashima, D. Nesvorný, A. Nguyen, L. Nittler, M. Onose, A. Pack, C. Park, L. Piani, L. Qin, S. S. Russell, N. Sakamoto, M. Schönbächler, L. Tafla, H. Tang, K. Terada, Y. Terada, T. Usui, S. Wada, M. Wadhwa, R. J. Walker, K. Yamashita, Q.-Z. Yin, T. Yokoyama, S. Yoneda, E. D. Young, H. Yui, A.-C. Zhang, T. Nakamura, H. Naraoka, T. Noguchi, R. Okazaki, K. Sakamoto, H. Yabuta, M. Abe, A. Miyazaki, A. Nakato, M. Nishimura, T. Okada, T. Yada, K. Yogata, S. Nakazawa, T. Saiki, S. Tanaka, F. Terui, Y. Tsuda,

S. Watanabe, M. Yoshikawa, S. Tachibana, H. Yurimoto, Ryugu's nucleosynthetic heritage from the outskirts of the Solar System. *Sci. Adv.* **8**, eadd8141 (2022).

8. N. Dauphas, B. Marty, L. Reisberg, Molybdenum evidence for inherited planetary scale isotope heterogeneity of the protosolar nebula. *Astrophys. J.* **565**, 640 (2002).

9. C. Burkhardt, N. Dauphas, U. Hans, B. Bourdon, T. Kleine, Elemental and isotopic variability in solar system materials by mixing and processing of primordial disk reservoirs. *Geochim. Cosmochim. Acta* **261**, 145–170 (2019).

10. G. J. MacPherson, Calcium-aluminum-rich inclusions in chondritic meteorites. *Meteor. Cosmochem. Proc.* **1**, 139–179 (2014).

11. A. M. Davis, F. M. Richter, 1.10-Condensation and evaporation of solar system materials. *Treatise Geochem. (2nd Ed.)* **1**, 335–360 (2014).

12. G. A. Brennecka, C. Burkhardt, G. Budde, T. S. Kruijer, F. Nimmo, T. Kleine, Astronomical context of Solar System formation from molybdenum isotopes in meteorite inclusions. *Science* **370**, 837–840 (2020).

13. C. Burkhardt, T. Kleine, F. Oberli, A. Pack, B. Bourdon, R. Wieler, Molybdenum isotope anomalies in meteorites: Constraints on solar nebula evolution and origin of the Earth. *Earth Planet. Sci. Lett.* **312**, 390–400 (2011).

14. T. Stephan, A. M. Davis, Molybdenum isotope dichotomy in meteorites caused by s-process variability. *Astrophys. J.* **909**, 8 (2021).

15. L. Grossman, Condensation in the primitive solar nebula. *Geochim. Cosmochim. Acta* **36**, 597–619 (1972).

16. K. D. McKeegan, A. P. A. Kallio, V. S. Heber, G. Jarzebinski, P. H. Mao, C. D. Coath, T. Kunihiro, R. C. Wiens, J. Nordholt, R. W. Moses Jr, D. B. Reisenfeld, A. J. G. Jurewicz, D. S. Burnett, The oxygen isotopic composition of the Sun inferred from captured solar wind. *Science* **332**, 1528–1532 (2011).

17. K. D. McKeegan, M. Chaussidon, F. Robert, Incorporation of short-lived 10Be in a calcium-aluminum-rich inclusion from the Allende meteorite. *Science* **289**, 1334–1337 (2000).

18. D. V. Bekaert, M. Auro, Q. R. Shollenberger, M.-C. Liu, H. Marschall, K. W. Burton, B. Jacobsen, G. A. Brennecka, G. J. MacPherson, R. von Mutius, A. Sarafian, S. G. Nielsen, Fossil records of early solar irradiation and cosmolocation of the CAI factory: A reappraisal. *Sci. Adv.* **7**, eabg8329 (2021).

19. T. R. Ireland, B. Fegley Jr, The solar system's earliest chemistry: Systematics of refractory inclusions. *Int. Geol. Rev.* **42**, 865–894 (2000).

20. B. Mason, S. R. Taylor, "Inclusions in the Allende meteorite" (Smithsonian Contributions to the Earth Sciences, Smithsonian Institution Press, 1982).

21. T. Tanaka, A. Masuda, Rare-earth elements in matrix, inclusions, and chondrules of the Allende meteorite. *Icarus* **19**, 523–530 (1973).

22. J. Hu, N. Dauphas, F. Tissot, R. Yokochi, T. Ireland, Z. Zhang, A. Davis, F. Ciesla, L. Grossman, B. L. A. Charlier, M. Roskosz, E. E. Alp, M. Y. Hu, J. Zhao, Heating events in the nascent solar system recorded by rare earth element isotopic fractionation in refractory inclusions. *Sci. Adv.* **7**, eabc2962 (2021).

23. G. A. Brennecka, L. E. Borg, M. Wadhwa, Evidence for supernova injection into the solar nebula and the decoupling of r-process nucleosynthesis. *Proc. Natl. Acad. Sci. U.S.A.* **110**, 17241–17246 (2013).

24. Q. R. Shollenberger, J. Render, G. A. Brennecka, Er, Yb, and Hf isotopic compositions of refractory inclusions: An integrated isotopic fingerprint of the solar system's earliest reservoir. *Earth Planet. Sci. Lett.* **495**, 12–23 (2018).

25. G. Brennecka, S. Weyer, M. Wadhwa, P. E. Janney, J. Zipfel, A. D. Anbar, $^{238}U/^{235}U$ variations in meteorites: Extant 247Cm and implications for Pb-Pb dating. *Science* **327**, 449–451 (2010).

26. H. Hidaka, M. Ebihara, S. Yoneda, Isotopic study of neutron capture effects on Sm and Gd in chondrites. *Earth Planet. Sci. Lett.* **180**, 29–37 (2000).

27. T. Stephan, R. Trappitsch, P. Hoppe, A. M. Davis, M. Bose, A. Boujibar, F. Gyngard, K. M. Hynes, N. Liu, L. R. Nittler, The Presolar Grain Database. I. Silicon carbide. *Astrophys. J. Suppl. Ser.* **270**, 27 (2024).

28. S. Bisterzo, R. Gallino, O. Straniero, S. Cristallo, F. Käppeler, The s-process in low-metallicity stars–II. Interpretation of high-resolution spectroscopic observations with asymptotic giant branch models. *Mon. Not. R. Astron. Soc.* **418**, 284–319 (2011).

29. A. M. Davis, J. Zhang, N. D. Greber, J. Hu, F. L. Tissot, N. Dauphas, Titanium isotopes and rare earth patterns in CAIs: Evidence for thermal processing and gas-dust decoupling in the protoplanetary disk. *Geochim. Cosmochim. Acta* **221**, 275–295 (2018).

30. R. E. Lingenfelter, E. H. Canfield, W. N. Hess, The lunar neutron flux. *J. Geophys. Res.* **66**, 2665–2671 (1961).

31. I. Leya, J. Masarik, Thermal neutron capture effects in radioactive and stable nuclide systems. *Meteor. Planet. Sci.* **48**, 665–685 (2013).

32. N. Dauphas, A. M. Davis, B. Marty, L. Reisberg, The cosmic molybdenum–ruthenium isotope correlation. *Earth Planet. Sci. Lett.* **226**, 465–475 (2004).

33. N. Dauphas, J. H. Chen, J. Zhang, D. A. Papanastassiou, A. M. Davis, C. Travaglio, Calcium-48 isotopic anomalies in bulk chondrites and achondrites: Evidence for a uniform isotopic reservoir in the inner protoplanetary disk. *Earth Planet. Sci. Lett.* **407**, 96–108 (2014).

34. X. Chen, N. Dauphas, Z. J. Zhang, B. Schoene, M. Barboni, I. Leya, J. Zhang, D. Szymanowski, K. D. McKeegan, Methodologies for ^{176}Lu–^{176}Hf analysis of Zircon grains from the Moon and beyond. *ACS Earth Space Chem.* **8**, 36–53 (2023).

35. Q. R. Shollenberger, L. E. Borg, J. Render, S. Ebert, A. Bischoff, S. S. Russell, G. A. Brennecka, Isotopic coherence of refractory inclusions from CV and CK meteorites: Evidence from multiple isotope systems. *Geochim. Cosmochim. Acta* **228**, 62–80 (2018).

36. B. Charlier, F. L. H. Tissot, N. Dauphas, C. J. N. Wilson, Nucleosynthetic, radiogenic and stable strontium isotopic variations in fine-and coarse-grained refractory inclusions from Allende. *Geochim. Cosmochim. Acta* **265**, 413–430 (2019).

37. T. Hopp, G. Budde, T. Kleine, Heterogeneous accretion of Earth inferred from Mo-Ru isotope systematics. *Earth Planet. Sci. Lett.* **534**, 116065 (2020).

38. B. L. A. Charlier, F. L. Tissot, H. Vollstaedt, N. Dauphas, C. J. N. Wilson, R. T. Marquez, Survival of presolar p-nuclide carriers in the nebula revealed by stepwise leaching of Allende refractory inclusions. *Sci. Adv.* **7**, eabf6222 (2021).

39. C. Clarke, G. Lodato, S. Y. Melnikov, M. A. Ibrahimov, The photometric evolution of FU Orionis objects: disc instability and wind—envelope interaction. *Mon. Not. R. Astron. Soc.* **361**, 942–954 (2005).

40. L. Cacciapuoti, L. Testi, L. Podio, C. Codella, A. Maury, M. De Simone, P. Hennebelle, U. Lebreuilly, R. Klessen, S. Molinari, Protostellar chimney flues: Are jets and outflows lifting submillimeter dust grains from disks into envelopes? *Astrophys. J.* **961**, 90 (2024).

41. Q. R. Shollenberger, J. Render, M. K. Jordan, K. A. McCain, S. Ebert, A. Bischoff, T. Kleine, E. D. Young, Titanium isotope systematics of refractory inclusions: Echoes of molecular cloud heterogeneity. *Geochim. Cosmochim. Acta* **324**, 44–65 (2022).

42. L. Kööp, A. M. Davis, D. Nakashima, C. Park, A. N. Krot, K. Nagashima, T. J. Tenner, P. R. Heck, N. T. Kita, A link between oxygen, calcium and titanium isotopes in ^{26}Al-poor hibonite-rich CAIs from Murchison and implications for the heterogeneity of dust reservoirs in the solar nebula. *Geochim. Cosmochim. Acta* **189**, 70–95 (2016).

43. S. Krijt, S. Arakawa, M. Oosterloo, H. Tanaka, A closer look at individual collisions of dust aggregates: Material mixing and exchange on microscopic scales. *Mon. Not. R. Astron. Soc.* **534**, 2125–2133 (2024).

44. A. J. Cridland, G. P. Rosotti, B. Tabone, Ł. Tychoniec, M. McClure, P. Nazari, E. F. van Dishoeck, Early planet formation in embedded protostellar disks - Setting the stage for the first generation of planetesimals. *Astron. Astrophys.* **662**, A90 (2022).

45. E. T. Dunham, A. Sheikh, D. Opara, N. Matsuda, M.-C. Liu, K. D. McKeegan, Calcium–aluminum-rich inclusions in non-carbonaceous chondrites: Abundances, sizes, and mineralogy. *Meteor. Planet. Sci.* **58**, 643–671 (2023).

46. F. L. H. Tissot, N. Dauphas, L. Grossman, Origin of uranium isotope variations in early solar nebula condensates. *Sci. Adv.* **2**, e1501400 (2016).

47. J. Y. Hu, F. L. Tissot, R. Yokochi, T. J. Ireland, N. Dauphas, H. M. Williams, Determination of rare earth element isotopic compositions using sample-standard bracketing and double-spike approaches. *ACS Earth Space Chem.* **7**, 2222–2238 (2023).

48. D. York, N. M. Evensen, M. L. Martínez, J. D. B. Delgado, Unified equations for the slope, intercept, and standard errors of the best straight line. *Am. J. Phys.* **72**, 367–375 (2004).

49. A. Bouvier, M. Boyet, Primitive Solar System materials and Earth share a common initial ^{142}Nd abundance. *Nature* **537**, 399–402 (2016).

50. E. Anders, N. Grevesse, Abundances of the elements: Meteoritic and solar. *Geochim. Cosmochim. Acta* **53**, 197–214 (1989).
